# Factors Associated With Kirschner Wire Backout After Tension Band Wiring for Olecranon Fractures: A Retrospective Study

**DOI:** 10.7759/cureus.103406

**Published:** 2026-02-11

**Authors:** Takahiro Maeda, Tomoyasu Homma, Misato Sakamoto, Hideaki Ishii, Shu Yoshizawa, Hiroyasu Ikegami

**Affiliations:** 1 Department of Orthopedic Surgery, Toho University Ohashi Medical Center, Tokyo, JPN

**Keywords:** intramedullary fixation, irritation, k-wire backout, olecranon fracture, reoperation, tension band wiring

## Abstract

Background: Tension band wiring (TBW) is widely used for olecranon fractures; however, posterior migration of Kirschner wires (K-wires), termed “backout,” is a common complication. At our institution, during intramedullary fixation, the proximal ends of K-wires are bent by 180° and embedded into the olecranon fragment. This study aimed primarily to identify clinical and radiographic factors associated with K-wire backout after TBW for olecranon fractures, and secondarily to explore clinically relevant thresholds of insertion depth and backout distance in relation to postoperative symptoms and reoperation.

Methods: We retrospectively reviewed data from 34 patients with olecranon fractures who underwent TBW and intramedullary K-wire fixation at our institute between 2014 and 2023. The backout distance was measured using postoperative radiographs. Patients were divided into backout (≥ 5 mm) and non-backout (< 5 mm) groups, and their clinical and radiographic parameters were compared.

Results: The mean follow-up period was 12 months. Irritation symptoms occurred in 11 (33%) of the cases, and implant removal was required in 18 (53%). The insertion depth was significantly shallower in the backout group than in the non-backout group. The data suggested that an insertion depth of approximately 4-5 mm may be a practical target to reduce the likelihood of clinically relevant backout. Although thinner K-wires (1.6 mm) were used more frequently in the backout group, diameter was not independently associated with backout. Backout exceeding approximately 7-8 mm was frequently observed in patients with irritation symptoms and those who underwent reoperation.

Conclusion: In this retrospective cohort, a shallower embedding depth of the bent K-wire tip was associated with a higher likelihood of postoperative backout. Ensuring sufficient insertion depth and the use of thicker wires, when feasible, may help reduce complications following TBW.

## Introduction

Olecranon fractures are one of the most common elbow injuries, accounting for approximately 10% of all upper extremity fractures [1,2]. These injuries typically result from falls or direct trauma and require stable fixation to allow early mobilization and prevent long-term functional impairment [2]. Given the importance of the elbow in activities of daily living, achieving both anatomical reduction and stable fixation is essential for good clinical outcomes.

Tension band wiring (TBW), first described by Weber et al. [3], has long been considered the standard surgical technique for simple displaced olecranon fractures. This method is based on the biomechanical principle of converting the tensile forces from the triceps into compressive forces at the fracture site, thereby promoting bone healing [1,4]. Several surgical options have been described for the management of displaced olecranon fractures, including TBW, plate fixation, and intramedullary fixation techniques, with the choice of treatment depending on fracture characteristics and patient-related factors [5]. TBW is widely used because of its technical simplicity, low invasiveness, and cost-effectiveness; however, recent clinical studies have questioned whether TBW should still be regarded as the preferred fixation method, particularly given its high rate of implant-related complications and reoperations [2,5].

Among these complications, posterior migration of Kirschner wires (K-wires), commonly referred to as “backout,” is one of the most frequent. Backout can lead to skin irritation, pain, and infection and often necessitates implant removal, contributing significantly to patient dissatisfaction and increased reoperation rates [6-8]. Reportedly, implant removal is more frequently required after TBW than after other fixation methods, highlighting the clinical burden of this complication [8].

Several technical modifications have been proposed to reduce the risk of backout, including changes in wire diameter, configuration, and insertion techniques [1,6,7]. However, reports remain inconsistent, and no consensus has been established regarding the optimal method to prevent migration. Moreover, most previous studies have focused on limited technical aspects, and comprehensive evaluations of clinical and radiographic factors associated with K-wire backouts remain scarce.

Therefore, identifying potentially modifiable risk factors for K-wire backout is clinically important, as such information may provide practical intraoperative guidance and help reduce postoperative implant-related complications. Accordingly, this retrospective study aimed to primarily identify clinical and radiographic factors associated with K-wire backout after TBW, with a particular focus on the intramedullary insertion depth of the bent K-wire tip. Additionally, we sought to explore clinically relevant thresholds of insertion depth and backout distance in relation to postoperative irritation symptoms and the need for reoperation. This study was designed as an exploratory, hypothesis-generating analysis.

## Materials and methods

We retrospectively reviewed data from 34 patients (14 men and 20 women) who underwent surgical treatment for olecranon fractures using TBW with intramedullary K-wire fixation at our institution between 2014 and 2023. In all cases, a standard tension band wiring technique was used. Two intramedullary K-wires were inserted from the olecranon tip, and the proximal ends were cut, bent by approximately 180°, and embedded into the olecranon fragment. No alternative fixation constructs (e.g., transcortical fixation or locked TBW) were used in this cohort.

Inclusion criteria were patients with acute olecranon fractures treated with tension band wiring using intramedullary K-wires at our institution. Exclusion criteria included pathological fractures, revision surgeries, open fractures requiring staged procedures, and cases with insufficient radiographic follow-up for measurement.

Preoperative imaging was used to classify the fracture types according to the Colton classification. Postoperative lateral elbow radiographs were obtained to measure the K-wire backout distance. To minimize the effect of radiographic magnification, all measurements were performed using the known diameter of the K-wire as an internal reference. Measurements were made along the longitudinal axis of the K-wire using standardized digital imaging software. All radiographic measurements were performed retrospectively by the authors using stored digital images. Interobserver and intraobserver reliability were not formally assessed, which may have affected the precision of radiographic measurements. For comparison of participant characteristics, backout distance was defined as the protrusion length of the K-wire measured from the dorsal cortical entry point of the olecranon to the bent proximal tip on lateral radiographs. Patients with a backout distance (protrusion length) ≥ 5 mm were categorized into the backout group (Group B, n = 18), while those with a backout distance < 5 mm were categorized into the non-backout group (Group N, n = 16). The 5-mm threshold was adopted as a pragmatic reference based on prior reports and surgical relevance, rather than as a validated clinical cutoff [7].

The following variables were analyzed: age, sex, intramedullary insertion depth of the bent K-wire, backout distance, distance from the K-wire tip to the articular surface, distance from the soft-wire hole to the fracture line, range of motion (ROM), bending angle of the K-wire, wire diameter (1.6 mm or 1.8 mm), total intramedullary length of the K-wire (KW length), presence of soft-wire loosening, irritation symptoms, hardware removal, and fracture type (Colton classification).

The KW length was assessed based on an anatomical study by Shuman et al. [9], in which the average position of the sagittal ulnar arch was defined as 51.8% of the total ulnar length (approximately 140.8 mm). Patients with KW lengths exceeding this threshold were assigned to the deep group (Group D), and those below this threshold were assigned to the proximal group (Group P).

Because of the retrospective design, certain procedural details, including minor variations in wire-bending technique, insertion angle, and postoperative rehabilitation, were not fully standardized.

Statistical analyses were performed using R software (version 4.2.2; R Development Core Team, Vienna, Austria). The Mann-Whitney U test, Fisher's exact test, Wilcoxon test, and receiver operating characteristic (ROC) curve analyses were used, with statistical significance set at p < 0.05. Given the small sample size, all analyses should be interpreted as exploratory. Subgroup and ROC analyses may be underpowered and prone to type I error. No a priori sample size or power calculation was performed.

## Results

The mean follow-up duration was 12 months. The mean patient age was 59 years (range: 13-89 years). The overall cohort comprised 18 type A, 12 type B, and four type C fractures. The mean ROM was 128.0° flexion and -6.9° extension. Bone union was achieved in all patients.

Irritation symptoms were observed in 11 (33%) of cases, and hardware removal was required in 18 (53%). In most cases, the K-wire tips were cut, bent by 180°, and buried within the olecranon. The mean backout distance was 6.3 ± 5.7 mm, and the mean intramedullary insertion depth of the bent wire was 2.2 ± 2.0 mm. Wires with diameters of 1.6 and 1.8 mm were used. A significant negative correlation was observed between insertion depth and backout distance (r = -0.60, p < 0.001).

**Figure 1:**
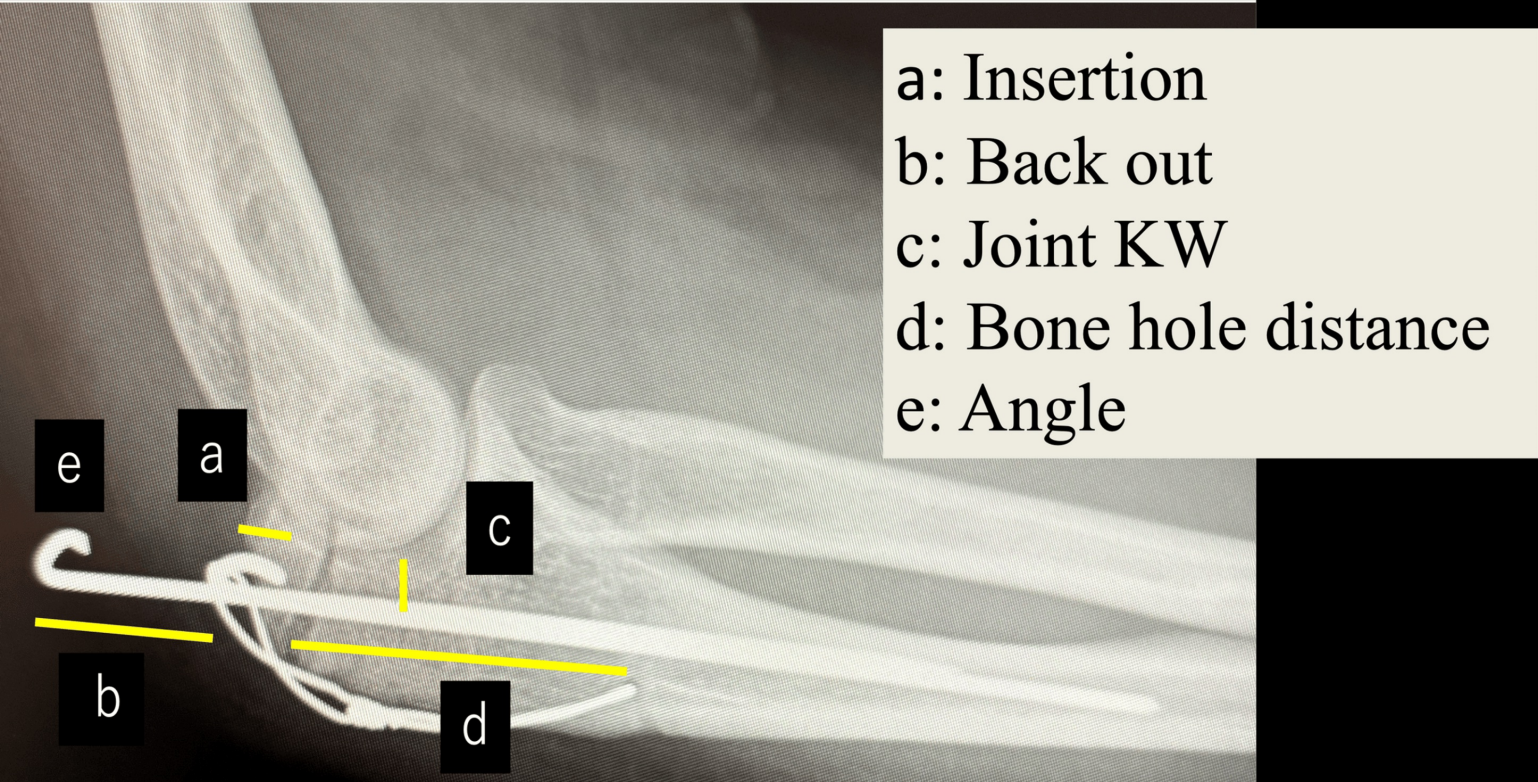
Measurement sites for each parameter in radiographs. The distances for each parameter were measured using X-ray images. (a) Length inserted into the bone at the bent end of the K-wire; (b) Backout distance, defined as the protrusion length measured from the dorsal cortical entry point of the olecranon to the bent proximal tip of the K-wire; (c) Distance from the olecranon joint surface to the K-wire; (d) Distance from the fracture line to the bone hole of the soft wire; (e) Bending angle of the K-wire end. K-wire: Kirschner wire

**Figure 2:**
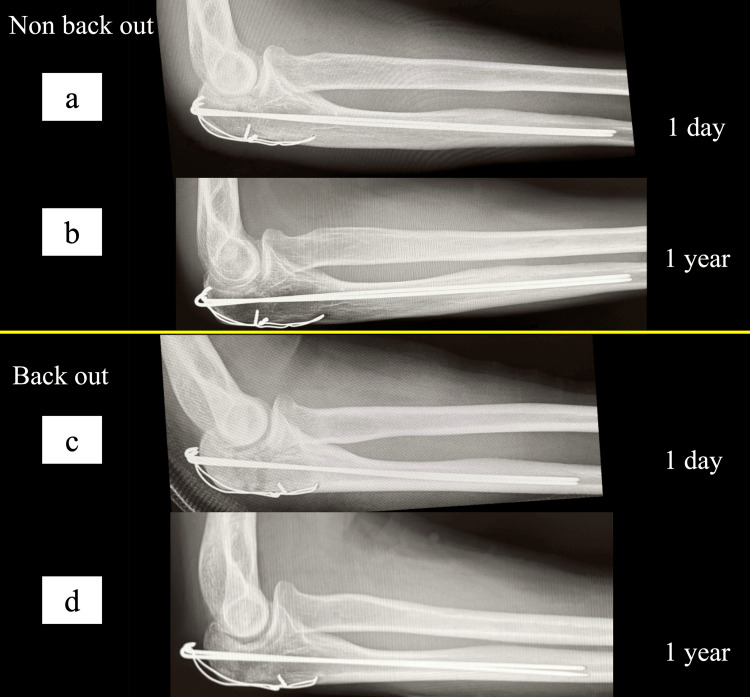
Representative cases of the K-wire backout. The upper panels show cases without the K-wire backout, and the lower panels show cases with the K-wire backout. (a, b) Non-backout case at one day (a) and one year (b) postoperatively. (c, d) Backout case at one day (c) and one year (d) postoperatively. K-wire: Kirschner wire

In intergroup comparison, the insertion depth was significantly smaller in Group B (2.0 ± 1.5 mm) than in Group N (4.3 ± 2.3 mm, p < 0.05). Thinner wires (1.6 mm) were used more frequently in Group B in univariate analysis. No significant difference in KW length was observed between the groups. However, irritation symptoms were significantly more common in Group B than in Group N. No significant differences were observed in ROM, articular surface distance, wire-hole position, soft wire loosening, or fracture type. Implant removal rates did not differ significantly between the groups (Tables 1-2, Figure 1). Representative cases demonstrating minimal backout in the non-backout group and marked posterior migration in the backout group are shown (Figure 2).

**Table 1:** Comparison of case numbers for each parameter between groups. Fisher’s exact test, *p < 0.05 SW: soft wire; KW length: total intramedullary length of the Kirschner wire; D: distal; P: proximal; HWR: hardware removal

Characterstic		Group B	Group N	p-value
Sex	Female	9	11	0.315
	Male	9	5	
K-wire	φ1.6mm	15	6	0.012*
	φ1.8mm	3	10	
KW length	D	8	5	0.429
	P	3	5	
SW loosening	(+)	6	2	0.233
	(-)	12	14	
Irritation	(+)	8	0	0.003*
	(-)	10	16	
HWR	(+)	11	7	0.492
	(-)	7	9	
Colton classification	Type A	7	11	0.109
	Type B	7	5	
	Type C	4	0	

**Table 2:** Comparison of clinical parameters between groups. Values are presented as mean ± standard deviation; Mann-Whitney U test. *p < 0.05 KW: Kirschner wire; ROM: range of motion; Angle: Bending angle at the end of the KW

Characterstic	Group B	Group N	p-value
Age (years)	61.2 ± 21.0	56.3 ± 20.5	0.447
a: Insertion (mm)	2.0 ± 1.5	4.3 ± 2.3	0.001*
b: Backout (mm)	10.3 ± 4.9	1.9 ± 1.9	<0.001*
c: Joint KW (mm)	5.3 ± 1.5	5.0 ± 1.4	0.442
d: Bone hole distance (mm)	30.6 ± 9.1	33.0 ± 10.0	0.528
e: Angle (°)	161.7 ± 13.4	162.6 ± 15.6	0.768
ROM flexion (°)	123.9 ± 15.8	133.0 ± 5.42	0.171
ROM extension (°)	-8.06 ± 6.04	-5.67 ± 4.42	0.275

In the multivariate logistic regression model, including K-wire diameter and insertion depth, insertion depth remained independently associated with backout ≥5 mm. Each 1-mm increase in insertion depth significantly reduced the risk of backout (odds ratio (OR): 0.59, 95% confidence interval (CI): 0.38-0.90; p = 0.013). K-wire diameter (1.6 mm vs. 1.8 mm) showed a trend toward association, with thinner wires being more likely to lead to backout (OR: 5.08, 95% CI: 0.86-29.9; p = 0.072); however, this relationship was not statistically significant (Table 3).

**Table 3:** Multivariate logistic regression analysis for factors associated with the K-wire backout (≥ 5 mm). *p < 0.05 OR: odds ratio; CI: confidence interval; K-wire: Kirschner wire

Characterstic	OR	95% CI (Lower-Upper)	p-value
K-wire diameter (1.6 mm vs 1.8 mm)	5.08	0.86-29.9	0.072
Insertion depth (per 1 mm increase)	0.59	0.38-0.90	0.013*

The Wilcoxon test revealed a significantly greater backout distance in patients with irritation symptoms (median: 12.78 mm). ROC analysis revealed a backout cutoff value of 7.93 mm for predicting irritation (sensitivity: 87.5%; specificity: 88.5%; area under the curve (AUC): 0.90). For hardware removal, the cutoff value was 7.23 mm (AUC: 0.71) (Table 4).

**Table 4:** Wilcoxon test and ROC analyses of the association between the backout distance and clinical outcomes. *p < 0.05 ROC: receiver operating characteristics; AUC: area under the curve; BD: backout distance, HWR: hardware removal

Analysis (with ROC)	Wilcoxon W	P value	AUC	Cut-off Value	Sensitivity	Specificity	Backout Median (Positive)	Backout Median (Negative)
BD vs Irritation	W = 188	P < 0.001*	0.90	7.93mm	87.5%	88.5%	12.78 mm	4.52 mm
BD vs HWR	W = 84	P = 0.040*	0.71	7.23mm	77.8%	69%	7.58 mm	4.52 mm

## Discussion

Olecranon fractures are among the most common upper extremity injuries, and TBW has long served as the standard surgical technique for simple displaced fractures [1,3]. However, K-wire backout, a frequent complication reported in 40-80% of cases, represents a major cause of postoperative pain, skin irritation, and reoperation [6,7]. Furthermore, studies investigating the long-term outcomes of TBW have indicated that the procedure is associated with a high rate of implant-related complications, raising the question of whether it should be regarded as the “gold standard,” particularly in younger and more active patients [2].

Previous studies have consistently reported a high incidence of implant-related complications following tension band wiring, particularly symptomatic K-wire prominence and the need for hardware removal [10]. Macko et al. first described these complications in a large clinical series, highlighting the inherent limitations of conventional TBW [10]. Saeed et al. further demonstrated that K-wire migration is influenced by technical factors, emphasizing the importance of proper wire placement and fixation technique [6]. Similarly, Chan et al. reported that K-wire positioning significantly affects postoperative complications and removal rates [11].

In this study, we demonstrated that a shallower embedding depth of the bent K-wire tip within the olecranon fragment (insertion depth) was the only independent risk factor for backout ≥ 5 mm. Multivariate analysis revealed that each additional millimeter of insertion reduced the risk of backouts by approximately 40%. Moreover, the median insertion depth was 1.93 mm in the backout group compared with 4.92 mm in the non-backout group, suggesting that an embedding depth of approximately 4-5 mm may be a reasonable target in clinical practice. This finding suggests that insertion depth may represent an important modifiable intraoperative factor associated with K-wire backout.

Furthermore, in the univariate analysis, the use of 1.6-mm wires was significantly associated with backout; however, this association disappeared after adjusting for insertion depth in the multivariate analysis, suggesting that the effect of wire diameter may be confounded by insertion depth. Nevertheless, previous studies have consistently reported that thicker wires provide greater stiffness and resistance to migration [1,7]; accordingly, the use of 1.8-mm wires should be considered whenever possible. Moreover, Villanueva et al. [1] have emphasized that inappropriate wire diameters and inadequate insertion techniques contribute to TBW failure, underscoring the potential importance of meticulous surgical techniques.

This study further explored the clinical relevance of the backout distance. ROC analysis demonstrated cutoff values of 7.9 mm (AUC: 0.90) for predicting irritation symptoms and 7.2 mm (AUC: 0.71) for predicting reoperation, suggesting that a protrusion length exceeding approximately 7-8 mm may be clinically relevant. Similarly, in a large multicenter study by Midtgaard et al. [8], symptomatic implant irritation was the leading cause of reoperation after TBW, with intramedullary K-wires increasing the risk of reoperation by more than fourfold. In addition to corroborating these findings, our study provides specific threshold values with potential clinical applicability. These ROC-derived values should be interpreted as descriptive, hypothesis-generating thresholds rather than externally validated predictors.

Herein, the total KW length did not significantly differ between groups; however, patients in whom wires extended beyond the sagittal bow of the ulna (approximately 52% of the total ulnar length), as described by Shuman et al. [9], tended to show less migration. Although this trend was not statistically significant, the sagittal bow may serve as a useful anatomical landmark for optimizing intraoperative intramedullary K-wire placement. Huang et al. [7] reported that wires confined to the proximal medullary canal were more prone to migration, whereas insertions extending further distally provided greater stability. Taken together with our results, these findings suggest that sufficient embedding, ideally extending beyond the sagittal bow, may be more effective in suppressing migration.

Recently, alternative surgical methods for the management of olecranon fractures have been proposed. Recent systematic reviews and meta-analyses have highlighted that conventional tension band wiring for olecranon fractures is associated with higher overall complication and implant removal rates compared with alternative fixation techniques, underscoring the ongoing need to optimize traditional TBW strategies [12]. Locked TBW, a modification of the conventional technique, has been reported to reduce complications and reoperation rates in a multicenter study [13]. Additionally, plate fixation has demonstrated favorable outcomes in complex fractures [1,5]. Studies assessing the long-term outcomes of these procedures have suggested that plate fixation and TBW yield similar functional results; however, TBW carries a higher risk of implant-related complications [5,6,14]. Recent clinical studies comparing TBW and plate fixation for simple olecranon fractures have reported comparable functional outcomes, although TBW tends to be associated with higher rates of implant-related complications and reoperation [15].

More recently, comparative studies and systematic reviews have suggested that alternative fixation methods, such as plate fixation, may reduce implant-related complications when compared with traditional TBW [16]. In addition, modified techniques, including locked tension band wiring, have been developed to address the high failure and reoperation rates associated with conventional methods [13]. Therefore, although TBW remains a commonly used option for simple fractures, ensuring an adequate insertion depth, selecting thicker wires when feasible, and tailoring fixation methods to the fracture type are essential for achieving optimal outcomes.

This study has some limitations. First, this was a single-center retrospective study; thus, it was subject to selection and information biases. Important confounders, such as surgeon experience, bone quality (including osteoporosis), fracture displacement severity, quality of reduction, and postoperative rehabilitation protocols, were not controlled for, and postoperative rehabilitation protocols were not standardized. Second, the relatively small sample size (n = 34) limited the statistical power, especially for subgroup analyses such as those for K-wire length or insertion beyond the sagittal bow. Third, surgeries were performed by multiple surgeons, which may have introduced variability in the insertion angle, bending method, and fixation strength. Fourth, although the mean follow-up period was 12 months, variability in follow-up duration among patients may have led to an underestimation of late migration or reoperation. In addition, this study did not include a comparison group treated with alternative fixation methods, such as plate fixation or locked tension band wiring, which limits direct comparison with other commonly used surgical techniques. Finally, radiographic measurements were performed retrospectively by the authors, and interobserver and intraobserver reliability were not assessed, which may limit the reproducibility and precision of the measurements. In addition, assessment of irritation symptoms was partly subjective, which may have introduced observer bias. Future studies should aim to validate our findings through large-scale multicenter prospective investigations. Such studies would allow for more robust subgroup analyses, minimize surgeon-dependent variability, and clarify the long-term clinical significance of insertion depth and backout distance in preventing complications after TBW.

## Conclusions

This study demonstrated that shallow intramedullary insertion and the use of thinner (1.6 mm) K-wires were associated with unfavorable postoperative outcomes after TBW. A backout distance exceeding approximately 8 mm was associated with irritation symptoms and hardware removal, suggesting a clinically relevant threshold.

To reduce the risk of backout-related complications, surgeons may consider embedding the ends of the K-wires into the bone, aiming for an insertion depth of approximately 4-5 mm, and prioritizing the use of 1.8 mm wires when feasible. Further prospective multicenter studies and direct comparisons with transcortical fixation are required to validate and generalize these findings.
